# Prognosis and Risk Factors of Radiation-Induced Lymphopenia in Early-Stage Lung Cancer Treated With Stereotactic Body Radiation Therapy

**DOI:** 10.3389/fonc.2019.01488

**Published:** 2020-01-24

**Authors:** Qianqian Zhao, Tingting Li, Gang Chen, Zhaochong Zeng, Jian He

**Affiliations:** Department of Radiation Oncology, Zhongshan Hospital, Fudan University, Shanghai, China

**Keywords:** radiation-induced lymphopenia, stereotactic body radiation therapy, early-stage lung cancer, prognosis, risk factors

## Abstract

**Background:** To investigate the role of stereotactic body RT (SBRT) in decreased total peripheral lymphocyte count (TLC) in patients with early-stage lung cancer and to explore possible risk factors for RT-induced lymphopenia.

**Materials and Methods:** We analyzed the TLCs and lymphocyte subsets of 76 patients in our prospective clinical database who received SBRT for early-stage lung cancer treatment. Relationships between clinical factors or dosimetric parameters and TLC were evaluated using Spearman's correlation analysis and Chi-square tests for continuous and categorical variables, respectively. Multivariate linear regression analysis was used to control for confounding factors. Kaplan–Meier analysis with a log-rank test and a multivariate Cox regression model were used for survival analysis.

**Results:** Most patients (64/76, 84.2%) experienced decreased absolute lymphocyte counts following SBRT, as well as shifts in lymphocyte subset distributions. Spearman's correlation coefficients between post-SBRT TLC and the percentage of the lung and heart receiving 5 to 50 Gy (in 5 Gy increments) shown that most lung DVH parameters [V(10)-V(50)] were significantly negatively correlated with post-SBRT TLC, while only heart V(5), V(20), V(25), V(30), and V(45) were significant. Univariate analyses revealed that a lower Pre-SBRT TLC level, higher mean lung dose, longer treatment duration, and longer TBT were significantly associated with a lower Post-SBRT TLC level (all *P* < 0.05). Stepwise multivariate linear regression, which incorporated all of the significantly clinical variables and SBRT-related parameters in univariate analysis, revealed that lower pre -SBRT TLC (*P* < 0.001), higher heart V5 (*P* = 0.002), and longer total beam-on time (TBT) (*P* = 0.001) were the independent risk factors for decrease in post-SBRT TLC. Patients with lower post-SBRT TLC and longer TBT exhibited significantly inferior progression-free survival (PFS) (*P* < 0.001 and *P* = 0.013) and overall survival (*P* = 0.006 and *P* = 0.043).

**Conclusions:** G2 and more severe lymphopenia after SBRT might be an independent prognostic factor for poorer outcome in early-stage lung cancer. Lowering heart V5 and TBT when designing SBRT plans may spare circulating lymphocytes and have the potential to further improve survival outcomes.

## Introduction

The immunological side effects of radiation therapy (RT) for tumor treatment are complex and include both stimulatory and inhibitory activity (1, 2). The enhancement of anti-tumor immunity by RT is best exemplified by the observation that RT could promote the death of tumor immunogenicity through the generation of an analogous *in situ* cancer vaccine (1, 3). Unfortunately, this positive impact is often counteracted by RT-induced lymphopenia (RIL) (4). Circulating lymphocyte populations are highly radiosensitive and can undergo apoptosis or depletion due to radiation exposure. Ultimately, RIL suppresses anti-tumor immunity and is associated with inferior survival in patients with various tumors, including lung cancer (5–10). Moreover, previous work has shown that RIL would further compromise the therapeutic efficacy of immune checkpoint inhibitors through the loss of effector cells, which identify and destroy tumor cells (11, 12). This further emphasized the importance of preserving and maintaining circulating lymphocytes in the setting of the new therapeutic strategy of combining radiotherapy (RT) and immunotherapy in cancer patients.

The degree of RIL depends on the RT total dose, target volume, and number of fractions given (13–16), although many prior studies of RIL have focused on conventional fractionated radiotherapy (CFRT) (5). Anti-tumor immunity alterations caused by stereotactic body radiation therapy (SBRT) and CFRT differ distinctly (17, 18). Until recently, however, comparatively little attention has been paid to SBRT-induced lymphopenia. In clinical practice, the significant effects of SBRT on the total peripheral lymphocyte count (TLC) and corresponding risk factors in patients with lung cancer have yet to be established.

Thus, we evaluated the role of SBRT in the reduction of the TLC in patients with lung cancer and explored possible risk factors of RIL. Based on our findings, we then offer some strategies for sparing peripheral lymphocytes and further improving prognoses of these patients.

## Materials and Methods

### Patient Eligibility and Clinical Characteristics

We analyzed our prospective clinical database of 171 patients who received definitive SBRT for lung cancer treatment between December 2014 and May 2018 at our institution. All patients underwent a comprehensive assessment before SBRT, including physical examination, laboratory analysis, chest computed tomography (CT) scans, abdominal CT or abdominal ultrasonography, brain magnetic resonance imaging, and bone scintigraphy. All patients with intrapulmonary tumors without pathological confirmation underwent ^18^F-fluorodeoxyglucose-positron emission tomography/computed tomography (^18^F-FDG PET/CT) scans. Diagnosis and treatment of these lesions were determined by a multidisciplinary lung cancer tumor team.

We applied the following study inclusion criteria for participant selection: (1) clinical early-stage lung cancer (tumor size <5 cm) without regional lymph metastasis [N0] and distant metastasis [M0]; (2) ≥ 18 years of age; (3) Karnofsky performance status (KPS) ≥ 70; (4) fewer than three pulmonary lesions treated with SBRT; (5) complete blood cell counts within 1 week before SBRT and within 1 week after completion of SBRT available; (6) peripheral total white blood cells (WBCs) above 2,000 cells/μl, and did not receive prophylactic or remedial treatment for decreased WBCs during SBRT treatment. Patients were excluded if they were pathologically diagnosed with small-cell lung cancer, were missing dosimetry data, had a history of other malignancy within the last 5 years, had received prior thoracic irradiation, or had acute or chronic inflammatory, hematologic, or systemic immune disorders.

The detailed procedures of SBRT administration for lung cancer at our institution have been described previously (19, 20). All patients were treated with SBRT using the Helical TomoTherapy Hi-Art treatment system (Accuray; Madison, WI, USA). The study protocol was approved by the ethics board of Zhongshan Hospital, Fudan University (Approval Number: B2014-128). All participants signed an informed consent form prior to study inclusion.

### Data Collection

The demographic and clinical information collected from participants included sex, age, KPS, smoking history, presence of respiratory system disease (chronic obstructive pulmonary disease, chronic bronchitis, or emphysema), tumor diameter, tumor location (central/peripheral), tumor histology, and total radiation dose. All laboratory values were measured using routine automated analyzers in the Clinical Laboratory of Zhongshan Hospital, Fudan University.

Venous blood samples were drawn from each patient at least twice: within 1 week before the start of SBRT (pre-SBRT) and within 1 week after completion of SBRT (post-SBRT) to quantify TLC and lymphocyte subset counts. Changes in the absolute counts and percentages of lymphocyte and lymphocyte subsets for each patient was calculated with the formula: Δvalue = post-SBRT value – pre-SBRT value. According to the National Cancer Institute Common Terminology Criteria for Adverse Events version 4.0, post-SBRT TLCs < 1,000 cells/μL were considered to indicate lymphopenia, and post-SBRT TLCs ≥ 1,000 cells/μL (G0) were non-lymphopenia. Lymphopenia was further categorized into grade 1 (G1, <1,000 cells/μL), grade 2 (G2, <800 cells/μL), grade 3 (G3, <500 cells/μL), and grade 4 (G4, <200 cells/μL). For analysis of cell numbers in blood, peripheral venous blood was collected in lavender top K3EDTA (ethylenediaminetetraacetic acid) collection tubes and stained directly with fluorochrome-conjugated monoclonal antibodies for 30 min at +4°C within 4 h of collection. The monoclonal antibodies used in this study were: FITC-labeled CD3, clone SK7; PE-labeled CD16, clone B73.1, and CD56, clone NCAM16.2; PerCP-Cy™5.5†-labeled CD45, clone 2D1 (HLe-1); PE-Cy™7-labeled CD4, clone SK3; APC-labeled CD19, clone SJ25C1;25 and APC-Cy7‡-labeled CD8, clone SK1. Following staining, red blood cells were lysed using FACS Lysing solution (BD Biosciences) and analyzed on the BD FACSanto™ Flow Cytometer (BDBiosciences) within 6 h of staining.

Dosimetric parameters were also extracted from the treatment planning system, including treatment duration, total beam-on time (TBT), gross tumor volume (GTV), planning target volume (PTV), mean lung dose (MLD), mean heart dose (MHD), and a wide range of dose-volume histogram (DVH) parameters for total lung and heart volume: V5, V10, V15, V20, V25, V30, V35, V40, V45, and V50. V*n* (%) corresponds to the percentage of total lung or heart volume receiving at least *n* dose of radiation. The treatment duration (days) of SBRT was defined as the number of days from SBRT start to SBRT completion. TBT (seconds) of SBRT was defined as the length of time of circulating lymphocyte exposure to radiation, which was calculated by beam-on time per fraction multiplied by fraction number.

### Patient Follow-Up and Outcomes

Follow-up duration and survival time were calculated from the start date of SBRT; the last follow-up date was May 30, 2019. Survival was censored if the patient was alive at the time of the last follow-up. Patients were generally evaluated weekly during SBRT, every 3 months following SBRT for the first 2 years, and every 6 months thereafter. PET/CT was performed only to distinguish recurrence from underlying SBRT-induced lung fibrosis. Progression-free survival (PFS) was calculated from the start date of SBRT to the date of any evidence of local or systemic cancer recurrence, death from any cause, or of the last follow-up. Overall survival (OS) was calculated from the start date of SBRT to the date of death from any cause or of the last follow-up.

### Statistical Analysis

Continuous variables were summarized as means ± standard deviation or medians (ranges) and compared using the Wilcoxon rank-sum test. Categorical variables were summarized as proportions and compared using Chi-square analysis or Fisher's exact test. Optimal cut-off values of continuous variables for survival prediction were determined based on the receiver-operating characteristic (ROC) curve (21). Relationships between clinical factors or dosimetric parameters and peripheral lymphocyte levels were evaluated using Spearman's correlation analysis for continuous variables and Chi-square tests for categorical variables. Spearman correlation coefficients were obtained for the association among different dosimetric parameters, then stepwise backward elimination with a selection criterion of *p* < 0.1 was applied to find the best subset of variables. Linear regression with a backward-forward stepwise method was used to analyze the relationships of the clinical variables and dosimetric parameters with post-SBRT TLC. The survival of patients with more than a 6-month follow-up time was analyzed further. The Kaplan–Meier estimator with a log-rank test was used to calculate and compare PFS and OS by patient covariates. Multivariate Cox regression with a backward-forward stepwise method was used to assess the potential influence of clinical factors and dosimetric parameters on PFS and OS. For multivariate linear and Cox regression analyses, potential variables with *P* < 0.1 in the univariate analysis were then included as covariates to identify their independent effect. *P*-values of <0.05 were considered statistically significant and reported as two-sided. All analyses were conducted using IBM SPSS statistical software (version 23, SPSS Inc.; Chicago, IL, USA).

## Results

### Patient Characteristics

**Figure 1:**
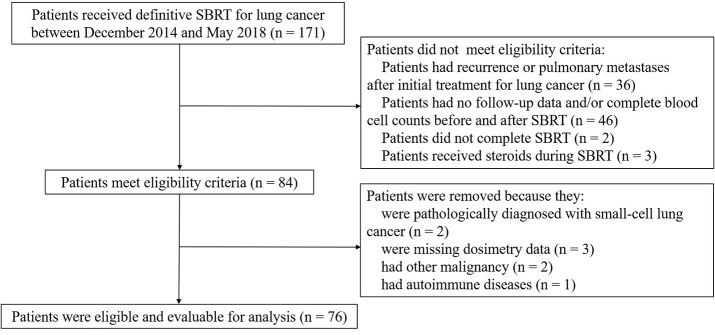
Identification of included and excluded patients with early-stage lung cancer receiving stereotactic body radiation therapy.

A total of 76 eligible patients with 81 small lung tumor lesions were enrolled in our study (Figure 1). The detailed characteristics of all of the patients are shown in Table 1.

**Table 1:** Baseline characteristics (*n* = 76).

**Characteristic**	***n* (%) or median (range)**
Sex	
Female	29 (38.16)
Male	47 (61.84)
Age at diagnosis (years)	72 (40–89)
Karnofsky performance status score	
≥80	55 (72.37)
<80	21 (27.63)
Smoking status	
Positive	32 (42.11)
Negative	44 (57.89)
Underlying respiratory system disease	
Yes	45 (59.21)
No	31 (40.79)
Tumor diameter (mm)	23.00 (9.00–48.00)
Gross tumor volume (cm^3^)	10.61 (0.64–85.37)
Tumor location	
Central	20 (26.32)
Peripheral	56 (73.68)
SUV_max_	5.10 (1.00–24.00)
Tumor histology	
Adenocarcinoma	39 (51.32)
Squamous	19 (25.00)
Unknown	18 (23.68)
SBRT dose and fractionation	
50 Gy in 5 fractions	26 (34.21)
60 Gy in 10 fractions	50 (65.79)

*SUV_max_, maximum standardized uptake value; SBRT, stereotactic body radiation therapy*.

### Changes in TLC and Lymphocyte Subset Counts Following SBRT

The gating strategy figures of one patient are shown in Supplementary Figure 1. Alterations of mean cell counts and percentages of total lymphocytes belonging to specific lymphocyte subsets following SBRT. Fifty-five patients had data on lymphocyte subsets available for analysis. As expected, the majority of patients (64/76, 84.2%) experienced decreased absolute lymphocyte counts following SBRT. The mean alteration of the peripheral lymphocyte count after SBRT was −526.04 cells/μL. In total, 27 (35.53%) patients developed lymphopenia. Of these, 13 (17.10%) developed G1 lymphopenia, 11 (14.47%) developed G2, and 3 (3.95%) developed G3. No patient experienced G4 lymphopenia. The percentages of all of the lymphocyte subsets tested were affected post-SBRT (all *P* < 0.05), including CD19^+^ B cells (fell by 53.88%), CD3^+^ T cells (by 30.56%), CD4^+^ T cells (by 34.64%), CD8^+^ T cells (by 25.96%), and CD56^+^ NK cells (by 13.28%). We observed a significant decrease in the CD19^+^ B cell percentage following SBRT from mean 10.85% to 7.23% (*P* < 0.001) and the CD4^+^ T cell percentage following SBRT from mean 37.95% to 36.27% (*P* = 0.031) and a significant increase in CD56^+^16 T cells from mean 20.95% to 24.70% (*P* < 0.001). No statistically significant differences were noted in alterations of other lymphocyte subset percentages (Table 2).

**Figure 2:**
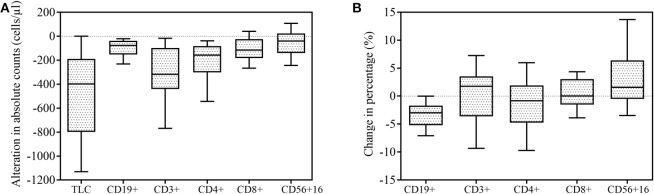
Effect of stereotactic body radiation therapy (SBRT) on peripheral lymphocyte counts (*n* = 76) and lymphocyte subsets (*n* = 55). All box-and-whisker plots show median (middle horizontal line), 75th percentile (top horizontal line), 25th percentile (bottom horizontal line), 90th percentile (top whisker), and 10th percentile (bottom whisker) for change in lymphocyte and lymphocyte subsets following SBRT. **(A)** Alteration in absolute counts of lymphocytes and lymphocyte subsets. **(B)** Percentage change in lymphocyte subsets.

**Table 2:** Mean ± standard deviation of peripheral lymphocyte count, lymphocyte subset counts, percentages of peripheral lymphocyte subsets, and CD4^+^/CD8^+^ before and after stereotactic body radiation therapy.

**Parameters**	***n***	**Pre-SBRT**	**Post-SBRT**	***P***
Total lymphocyte count (cells/μl)	76	1760.81 ± 649.06	1234.78 ± 528.82	< 0.001
CD19^+^ B count (cells/μl)	55	206.42 ± 133.86	95.00 ± 59.18	< 0.001
CD3^+^ T count (cells/μl)	55	1177.82 ± 522.81	818.00 ± 426.04	< 0.001
CD4^+^ T count (cells/μl)	55	682.76 ± 327.47	446.24 ± 226.61	< 0.001
CD8^+^ T count (cells/μl)	55	436.13 ± 238.54	322.91 ± 223.06	< 0.001
CD56^+^ NK count (cells/μl)	55	361.20 ± 269.13	313.25 ± 290.30	0.030
CD19^+^(%)	55	10.85 ± 4.70	7.23 ± 3.49	< 0.001
CD3^+^(%)	55	66.62 ± 13.17	66.28 ± 14.78	0.681
CD4^+^(%)	55	37.95 ± 9.60	36.27 ± 11.09	0.031
CD8^+^(%)	55	25.42 ± 10.31	25.83 ± 10.37	0.392
CD56^+^16(%)	55	20.95 ± 11.70	24.70 ± 14.58	< 0.001
CD4^+^/CD8^+^	55	1.81 ± 1.05	1.68 ± 0.96	0.017

*SBRT, stereotactic body radiation therapy; CD19^+^ B cells, B lymphocytes; CD3^+^ T cells, T lymphocytes; CD4^+^ T cells, T helper cells, CD8^+^ T cells, T cytotoxic cells; CD56^+^ NK cells, natural killer cells*.

### Correlations Between Post-SBRT TLC and Dosimetric Parameters

Spearman's correlation coefficients between post-SBRT TLC and the percentage of lung and heart receiving 5–50 Gy (in 5 Gy increments) are shown in Table 3. Most lung DVH parameters [V(10)-V(50) significantly negatively correlated with post-SBRT TLC, while only heart V(5), V(20), V(25), V(30), and V(45) were significant. Correlation coefficients remained greatest for lung V(50) (*r* = −0.337; *P* = 0.003) and heart V(25) (*r* = −0.362; *P* = 0.002). The correlation matrix among the different DVH parameters is presented in Supplementary Table 1.

**Table 3:** Correlation between post-SBRT total peripheral lymphocyte count and percentage of lung or heart dosed.

**Characteristic**	***n***	**Spearman correlation coefficient (r)**	***P*-value**
Lung V5	76	−0.204	0.076
Lung V10	76	−0.276	0.016
Lung V15	76	−0.261	0.023
Lung V20	76	−0.278	0.015
Lung V25	76	−0.287	0.012
Lung V30	76	−0.293	0.010
Lung V35	76	−0.282	0.014
Lung V40	76	−0.331	0.004
Lung V45	76	−0.284	0.013
Lung V50	76	−0.337	0.003
Heart V5	72	−0.235	0.047
Heart V10	72	−0.170	0.152
Heart V15	72	−0.217	0.067
Heart V20	72	−0.271	0.021
Heart V25	72	−0.362	0.002
Heart V30	72	−0.287	0.015
Heart V35	72	−0.221	0.062
Heart V40	72	−0.229	0.053
Heart V45	72	−0.307	0.009
Heart V50	72	−0.212	0.073

*SBRT, stereotactic body radiation therapy; Vn (%), the percentage of total lung or heart volume receiving at least n dose of radiation*.

### Association of Post-SBRT TLC With Clinical Factors

Univariate and multivariate linear regression analysis between characteristics and post-SBRT TLC levels are shown in Table 4. Univariate analyses revealed that higher Pre-SBRT TLC level, higher mean lung dose, longer treatment duration, and longer TBT were significantly associated with a lower Post-SBRT TLC level. Stepwise multivariate linear regression, which incorporated all significantly clinical variables and SBRT-related parameters in univariate analysis, showed that lower pre-SBRT TLC (*P* < 0.001), longer TBT (*P* = 0.001), and higher heart V5 (*P* = 0.002) were independent risk factors for decreased post-SBRT TLC.

**Table 4:** Univariate and multivariate linear regression analysis between characteristics and post-SBRT TLC.

**Characteristic**	**Regression coefficient**	**95% CI**	***P***
Univariate analysis			
Sex (female vs. male)	−32.517	−282.896 to 217.861	0.797
Age (year)	−3.266	−15.486 to 8.954	0.596
Karnofsky performance status (10%)	−3.030	−20.028 to 13.968	0.723
Smoker (smoker vs. never smoker)	−16.403	−231.169 to 263.976	0.895
Tumor diameter (mm)	−1.551	−14.304 to 11.203	0.809
Underlying respiratory system disease (yes vs. no)	97.891	−148.671 to 344.452	0.431
Pre-SBRT TLC (cells/μl)	0.528	0.385 to 0.672	<0.001
Dosimetric characteristics			
Gross tumor volume (cm^3^)	−1.125	−8.857 to 6.608	0.773
Planning target volume (cm^3^)	−1.995	−6.734 to 2.745	0.404
Mean lung dose (Gy)	−73.331	−139.641 to −7.021	0.031
Mean heart dose (Gy)	−34.819	−73.494 to 3.855	0.077
Radiation therapy			
Treatment duration (days)	−38.694	−69.801 to −7.587	0.015
Total beam-on time (seconds)	−0.129	−0.212 to −0.047	0.003
Fractionation (5 fractions vs. 10 fractions)	−215.285	−466.881 to 36.310	0.092
Multivariate analysis			
Pre-SBRT TLC (cells/μl)	0.524	0.393 to 0.656	< 0.001
Total beam-on time (seconds)	−0.103	−0.164 to −0.041	0.001
Heart V5	−5.452	−8.835 to −2.069	0.002

*SBRT, stereotactic body radiation therapy; TLC, total peripheral lymphocyte count; CI, confidence interval; V_n_, percentage of organ volume receiving n Gy*.

To evaluate if these associations existed pre-SBRT and were less likely to be SBRT-induced, we further assessed the relationships between pre-SBRT TLC and relevant patient characteristics (Table 5). Unlike post-SBRT TLC, we saw no significant differences in pre-SBRT TLC by sex, age, KPS, smoking status, underlying respiratory system disease, tumor diameter, tumor location, and SBRT-related parameters (all *P* > 0.05).

**Table 5:** Relationships of Pre-SBRT TLC levels with baseline characteristics in patients with early-stage lung cancer.

**Characteristic**	**Pre-SBRT lymphocyte count ≤ 1,600 (*n* = 39)**	**Pre-SBRT lymphocyte count > 1,600 (*n* = 37)**	***P* value**
Sex			
Male	27	20	
Female	12	17	0.173
Age (year)	74 (41–89)	70 (40–89)	0.189
Karnofsky performance status score			
≥80	29	26	
<80	10	11	0.690
Smoking status			
Positive	13	14	
Negative	26	18	0.368
Underlying respiratory system disease			
Yes	24	18	
No	15	19	0.259
Tumor diameter	23.00 (9.50–46.00)	23 (9.00–48)	0.686
Tumor location			
Central	7	13	
Peripheral	32	24	0.089
Dosimetric characteristics			
Gross tumor volume (cm^3^)	12.95 (0.64–62.36)	10.40 (0.67–85.37)	0.776
Planning target volume (cm^3^)	31.61 (4.14–105.35)	22.94 (3.82–116.20)	0.834
Mean lung dose (Gy)	4.38 (1.73–9.66)	4.27 (2.25–8.87)	0.719
Radiation therapy			
Treatment duration (days)	13 (5–16)	12 (5–20)	0.307
Irradiation time (seconds)	3599.00 (1921.50–8671.00)	3654.00 (1208.50–6384.00)	0.527
SBRT dose and fractionation			
50 Gy in 5 fractions	12	14	
60 Gy in 10 fractions	27	23	0.516

*SBRT, stereotactic body radiation therapy; TLC, total peripheral lymphocyte count*.

### Prognostic Value of Post-SBRT TLC

Survival analysis was performed to identify whether post-SBRT TLC exerted an independent prognostic influence on our patient population. Based on follow-up criteria, 63 patients were available for survival analysis. The median follow-up time was 22 months (range 6–55 months) for these patients, and at the end of the follow-up period, 53 (84.13%) patients were alive. In subgroup analysis, PFS and OS were not different between patients with G1 lymphopenia and those with G0 (*P* = 0.466 and *P* = 0.449, respectively). However, PFS and OS for G2-3 patients were significantly worse compared to G0-1 patients (*P* < 0.001 and *P* = 0.006, respectively). Considering this difference, we decided to classify patients into a G0-1 group and a G2-3 group to evaluate the prognostic value of post-SBRT TLC. In addition, we classified patients into a short TBT group (≤ 3,500 s) or high TBT group (>3,500 s) based on the ROC curve to evaluate the prognostic value of the beam-on time.

**Figure 3:**
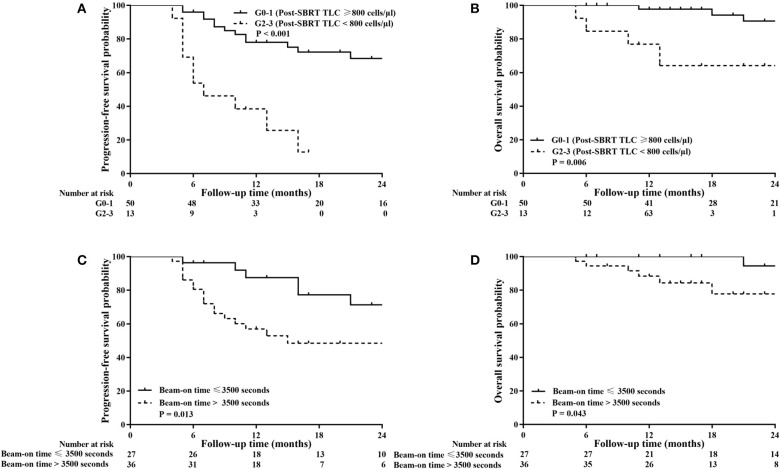
Kaplan–Meier analysis of progression-free survival (PFS) and overall survival (OS) stratified by post-SBRT lymphocyte counts **(A,B)** and total beam-on time **(C,D)**. High total peripheral lymphocyte count (TLC) following SBRT and short total beam-on time were significantly associated with improved PFS (*P* < 0.001 and *P* = 0.013) and OS (*P* = 0.006 and *P* = 0.043).

As shown in Figure 3, G0-1 and shorter TBT were significantly associated with improved PFS (*P* < 0.001 and *P* = 0.013) and OS (*P* = 0.006 and *P* = 0.043). Table 6 presents univariate and multivariate analysis results for PFS and OS including relevant variables. Multivariate analysis showed that G0-1 was significantly associated with improved PFS (hazard ratio [HR]: 0.183; 95% CI: 0.076 to 0.441; *P* < 0.001) and OS (HR: 0.169; 95% CI: 0.043 to 0.665; *P* = 0.011) and longer TBT was significantly associated with inferior PFS (HR: 3.066; 95% CI: 1.186 to 7.929; *P* = 0.021) after controlling for confounding variables.

**Table 6:** Cox regression analysis for progression-free survival and overall survival.

**Characteristic**	**Progression-free survival**		**Overall survival**	
	**HR (95% CI)**	***P***	**HR (95% CI)**	***P***
Univariate associations				
Sex				
Female				
Male	0.932 (0.413–2.103)	0.866	0.552 (0.142–2.140)	0.390
Age (years)				
≤ 70				
>70	1.159 (0.495–2.713)	0.734	2.557 (0.540–12.104)	0.237
KPS score				
<80				
≥80	0.816 (0.321–2.073)	0.669	0.300 (0.079–1.130)	0.075
Smoking status				
Positive				
Negative	0.530 (0.197–1.425)	0.208	1.313 (0.329–5.239)	0.700
Underlying respiratory system disease				
Yes				
No	0.734 (0.328–1.641)	0.451	0.460 (0.129–1.641)	0.231
Tumor location				
Central				
Peripheral	1.830 (0.810–4.132)	0.146	2.421 (0.695–8.429)	0.165
Tumor diameter (mm)				
≤ 30				
>30	1.006 (0.961–1.053)	0.799	0.625 (0.078–5.036)	0.659
SBRT dose and fractionation				
50 Gy in 5 fractions				
60 Gy in 10 fractions	0.735 (0.304–1.774)	0.494	1.175 (0.878–1.572)	0.277
Treatment duration (days)				
≤ 7				
>7	1.819 (0.678–4.880)	0.235	43.621 (0.189–9616.973)	0.194
Beam-on time (seconds)				
≤ 3,500				
>3,500	3.034 (1.194–7.708)	0.020	4.402 (0.922–21.022)	0.063
Pre-SBRT lymphocytes (cells/μl)				
≤ 1,600				
>1,600	1.223 (0.547–2.733)	0.623	1.587 (0.447–5.642)	0.475
Post-SBRT lymphocytes (cells/μl)				
<800 (G2-3)				
≥800 (G0-1)	0.187 (0.080–0.439)	< 0.001	0.178 (0.046–0.695)	0.013
Multivariate associations				
KPS score				
<80				
≥80	NI		0.281 (0.074–1.068)	0.062
Beam-on time (seconds)				
≤ 3,500				
>3,500	3.066 (1.186–7.929)	0.021	NI	
Post-SBRT lymphocytes (cells/μl)				
<800 (G2-3)				
≥800 (G0-1)	0.183 (0.076–0.441)	<0.001	0.169 (0.043–0.665)	0.011

*PFS, progression-free survival; OS, overall survival; HR, hazard ratio; CI, confidence interval; KPS, Karnofsky performance status; NI, not included in the multivariate model; SBRT, stereotactic body radiation therapy*.

## Discussion

The key observations from the present study include the following findings. First, the paired analysis complete blood counts pre- and post-SBRT for lung cancer revealed that patients experienced a substantially reduced circulating TLC (1760.81 ± 649.06 vs. 1234.78 ± 528.82; *P* < 0.001), despite the small radiation field. This finding is in accordance with other studies (14, 22). Second, our multivariate linear regression showed that lower pre-SBRT TLC, higher heart V5, and longer TBT were independent risk factors of RIL. Third, multivariate Cox proportional hazard regression models further identified that post-SBRT TLC and TBT were independently correlated with PFS and OS in our patient population.

Figure 2 illustrates changes in the lymphocyte subset distribution following SBRT due to unequal decreases in various subsets. Peripheral lymphocyte homeostasis was disturbed by SBRT, as both the absolute number and percentage of CD4^+^ T cells were significantly decreased after SBRT. Unlike CD4^+^ T cells, the absolute number of CD8^+^ T cells dropped less, and its relative percentage was nearly unchanged. Thus, the ratio of CD4^+^/CD8^+^ T cells decreased following SBRT (*P* = 0.017), which was also observed by Yang and colleagues in patients with head and neck cancer after receiving RT (23), although the radiosensitivities of CD4^+^ T and CD8^+^ T cells have been demonstrated to be similar (24). This result may be partially explained by SBRT's ability to promote priming and strong mobilization of CD8^+^ T cells, therefore compensating for the reduced absolute number of CD8^+^ T cells. This finding also supports the possibility that SBRT increases CD8^+^ T cell accumulation in tumor sites because the therapeutic efficacy of local ablative radiation critically depends on the presence of effector CD8^+^ T cells, but not CD4^+^ T cells (25–27).

An effective anti-tumor immune response requires functional lymphocytes capable of detecting and destroying tumor cells. Given that the majority of our patients developed severe RIL following SBRT, which impedes anti-tumor immunity, determining possible risk factors for RIL is important. Accumulated data indicate that RIL depends on irradiation volume and fraction number (14, 15), although these two aspects of SBRT were not identified as independent risk factors for RIL in the present study. Perhaps the irradiation volumes of our patients were too small to achieve statistical significance, unlike the larger target volume of patients with advanced lung cancer. However, multivariate analyses of possible risk factors in previous studies did not incorporate treatment duration and TBT as variables. In contrast, we included clinical variables and SBRT-related parameters (lung and heart DVH parameters, treatment duration, and TBT) and only identified pre-SBRT TLC, heart V5, and TBT as independent risk factors for RIL. Thus, we inferred that higher heart V5 and longer TBT contribute to RIL in lung cancer patients and should be considered when designing SBRT regimens so as to maximize the number of circulating lymphocytes sustained during irradiation treatment. In addition, a positive correlation between tumor volume and beam-on time was observed (*r* = 0.503, *P* < 0.001) in our study. We also conducted univariate and multivariate Cox regression analyses to assess the correlation between survival outcomes and tumor volume as well as beam-on time. No significant correlation was found between tumor diameter and survival outcomes (*P*-value was 0.799 for PFS and 0.659 for OS), while the beam-on time had a negative effect on survival outcomes, as shown in Table 6. These results suggest that shortening the beam-on time may spare peripheral lymphocytes and ultimately improve patient prognosis. Of course, further large-scale validation studies are needed to confirm the impact of beam-on time on lymphocyte populations in patients with NSCLC who receive SBRT.

The mechanism of RIL is not completely understood, although circulating lymphocytes in peripheral vessels are directly killed as they pass through radiation treatment fields (28). Because larger radiation fields and longer TBT expose circulating lymphocytes to more radiation, the reduction in TLC should be proportional to the target volume and TBT (14, 16, 29), a supposition supported by our results. Irradiation of bone marrow or lymphatic tissue may also cause direct destruction of lymphocytes. Apart from direct toxicity, irradiation may indirectly affect circulating lymphocyte levels via cytokine modulation (15). For example, interleukin-7 (IL-7), a key cytokine involved in T-cell proliferation, is essential for maintaining circulating T-cell homeostasis. Although its circulating level negatively correlates with CD4^+^ T cell counts (30), no compensatory rise in IL-7 levels in patients with severe RIL has been observed (31). Peiwen et al. reported an alternative cellular mechanism driving RIL related to the direct toxicity of radiation in SBRT-treated early-stage lung cancer. They considered that SBRT was delivered in a few fractions, thus limiting circulating lymphocyte exposure to ionizing irradiation as they pass through small radiation fields (32). However, SBRT was delivered with high ablative doses, as the biologically effective dose is often higher than 100 Gy. A negative correlation between the total radiation dose and post-RT TLC has also been demonstrated (29). Twelve patients in our study did not experience a decrease in peripheral lymphocytes. In this subset of our patient population, we speculate on whether the immune-stimulating effects of SBRT are greater than immunosuppressive effects or if their consistent TLC levels are driven by an unknown mechanism. Multiple questions and issues related to our observations remain unresolved: (1) the comprehensive effects of the target volume, fraction regimen, and total dose on RIL need to be explored; (2) the mechanism of lymphopenia development and its regulation needs to be characterized; (3) the optimal RT regimen to spare circulating lymphocytes need to be established. Given the clinical importance of this condition but the limited data regarding its nature and progression, additional research in this area is warranted. Several limitations should be considered in the interpretation of our findings. First, this study analyzed a single-centered dataset with limited patient numbers, so some useful predictors of RIL may have gone undetected. Second, several patients did not have pathological confirmation of pulmonary nodules because of the difficulty or perceived risk of obtaining small lesion specimens. However, all patients underwent ^18^F-FDG PET/CT scans, and the diagnosis and treatment options for these lesions were determined by a multidisciplinary tumor board. Third, complete blood counts were measured at only two time points: before and after SBRT; our database did not document consecutive circulating lymphocyte count changes. We could not definitively determine when levels of circulating lymphocytes began to recover following SBRT, although we plan to further investigate this aspect of TLC development. Finally, the population in our study is a little heterogenous, in that patients with a central tumor or tumor close to the rib received 60 Gy in 10 fractions while patients with peripheral tumors received 50 Gy in 5 fractions. Therefore, these results require further investigations in larger prospective trials for validation.

Despite these limitations, we demonstrated that G2 and more severe lymphopenia after SBRT might be an independent prognostic factor for poorer outcome in early-stage lung cancer. The data further suggested that lowering heart V5 and reducing TBT may spare circulating lymphocytes in this patient population. Specifically, limiting the heart radiation dose and TBT when designing SBRT regimens may be crucial for reducing lymphocyte radiotoxicity and improving patient survival, especially in patients with a relatively low pre-SBRT TLC level.

## Data Availability Statement

The datasets generated for this study are available on request from the corresponding author.

## Ethics Statement

The studies involving human participants were reviewed and approved by the ethics board of Zhongshan Hospital, Fudan University (B2014–128). The patients/participants provided their written informed consent to participate in this study.

## Author Contributions

ZZ and JH designed the study. QZ, TL, and GC contributed to the data collection. QZ analyzed the data and wrote the manuscript. All authors approved the version of the manuscript to be published.

### Conflict of Interest

The authors declare that the research was conducted in the absence of any commercial or financial relationships that could be construed as a potential conflict of interest.

## Abbreviations

RIL: RT-induced lymphopenia
CFRT: conventional fractionated radiotherapy
TLC: total peripheral lymphocyte count
TBT: total beam-on time
MLD: mean lung dose
MHD: mean heart dose.
