# Supplementary figures and images for: Prognosis and Risk Factors of Radiation-Induced Lymphopenia in Early-Stage Lung Cancer Treated With Stereotactic Body Radiation Therapy
Source: Front Oncol. 2020 Jan 24;9:1488. doi: 10.3389/fonc.2019.01488 (PMC6993213; doi:10.3389/fonc.2019.01488)

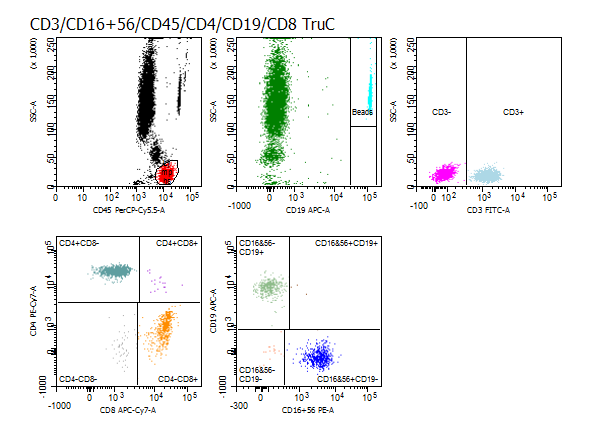

Supplement: Supplementary file 2 [file Image_1.tif]
